# Monitoring Butterfly Abundance: Beyond Pollard Walks

**DOI:** 10.1371/journal.pone.0041396

**Published:** 2012-07-30

**Authors:** Jérôme Pellet, Jason T. Bried, David Parietti, Antoine Gander, Patrick O. Heer, Daniel Cherix, Raphaël Arlettaz

**Affiliations:** 1 A.Maibach Sàrl, Oron-la-Ville, Switzerland; 2 Division of Conservation Biology, Institute of Ecology and Evolution, University of Bern, Bern, Switzerland; 3 Department of Zoology, Oklahoma State University, Stillwater, Oklahoma, United States of America; 4 Department of Ecology and Evolution, Biophore, University of Lausanne, Dorigny, Switzerland; 5 Groupe d’étude et de gestion, Grande Cariçaie, Champ-Pittet, Yverdon, Switzerland; 6 Musée de zoologie de Lausanne, Lausanne, Switzerland; Helmholtz Centre for Environmental Research - UFZ, Germany

## Abstract

Most butterfly monitoring protocols rely on counts along transects (Pollard walks) to generate species abundance indices and track population trends. It is still too often ignored that a population count results from two processes: the biological process (true abundance) and the statistical process (our ability to properly quantify abundance). Because individual detectability tends to vary in space (e.g., among sites) and time (e.g., among years), it remains unclear whether index counts truly reflect population sizes and trends. This study compares capture-mark-recapture (absolute abundance) and count-index (relative abundance) monitoring methods in three species (*Maculinea nausithous* and *Iolana iolas*: Lycaenidae; *Minois dryas*: Satyridae) in contrasted habitat types. We demonstrate that intraspecific variability in individual detectability under standard monitoring conditions is probably the rule rather than the exception, which questions the reliability of count-based indices to estimate and compare specific population abundance. Our results suggest that the accuracy of count-based methods depends heavily on the ecology and behavior of the target species, as well as on the type of habitat in which surveys take place. Monitoring programs designed to assess the abundance and trends in butterfly populations should incorporate a measure of detectability. We discuss the relative advantages and inconveniences of current monitoring methods and analytical approaches with respect to the characteristics of the species under scrutiny and resources availability.

## Introduction

Assessing species abundance is a fundamental requirement in ecology and conservation [1], [2]. Conservation practitioners must try to accurately detect patterns and trends in population abundance in order to set priorities for conservation action [3]. As it is impractical to survey all taxa, conservationists typically focus on groups that are expected to reflect broader biodiversity patterns, ecological change, or ecosystem integrity. This “coarse-filter” approach typically relies on plants or vertebrates as surrogates or umbrellas for insects and other invertebrates, but evidence is mixed on whether indirect conservation of invertebrates is effective (e.g., [4], [5]).

Butterflies and day-flying moths are widely believed to be reliable sentinels of environmental variation and human disturbance, with changes in distribution and abundance mirroring landscape, habitat and climate change [6]–[11]. Both species richness and species abundance estimates for butterflies usually rely on fixed-route transects (or Pollard walks, see [12], [13], [14]). The approach proposed in the early 1970’s by Ernie Pollard has become widely adopted and is the basis of many monitoring schemes [10], [15] around the world. Pollard walks entail counting butterflies along transects on a regular basis (e.g., weekly) throughout the flight season. These counts are then aggregated for each site (e.g., using the sum of the average weekly counts) to produce a species-specific abundance index [16]; sometimes the maximum count is used as the index.

Researchers have long wondered about the relationship between butterfly counts (and the aggregate index) and absolute population sizes. Douwes [17] tested this link by comparing estimates of absolute population size (obtained by capture-mark-recapture – CMR) and counts for *Boloria selene* and *Lycaena hippothoe*. Pollard [12], in his seminal paper, tested for a similar relationship using two satyrids (*Aphantopus hyperanthus* and *Coenonympha pamphilus*), and Thomas [18] compared CMR and count data for six additional species. These European studies, involving a wide range of species and families, suggest a correlation between index counts and population estimates. They thus seem to validate the approach proposed by Pollard. Yet, all these authors recognized several caveats of this approach: the proportion of the population actually counted varies with habitat type, with the fraction of the habitat that was surveyed, with observer’s experience and with factors such as weather, time of day and species behavior. Indeed, recent studies have confirmed that shifts in diurnal and seasonal distribution, weather conditions, representativeness of transect routes, vegetation succession, the ability of observers to detect species, and species behavioural response to population density are all potential sources of detectability variations [19]–[24]. Without adjustments for individual detectability, counts may not be comparable across sites, species, or time, which creates a major impediment to efficient and reliable monitoring and to evidence-based conservation action.

Obvious as it may seem, field biologists rarely observe all individuals in an area or population of interest. Despite this reality [25], the notion of individual detectability is still too often ignored in data processing. Relative population abundance or counts (*C*) may be linked to absolute population abundance (*N*) via the individual detection probability (*p*); the formal expression is simply *C = Np* [26]. Most monitoring programs tend to adjust survey protocols in order to keep individual detectability (*p*) as constant as possible so that changes in *C* to a large degree reflect changes in *N*. Although most wildlife ecologists are well aware that their counts represent some unknown fraction of the true populations, few recognize that this fraction varies even under standardized survey conditions [27]. Monitoring programs that use Pollard walks to draw indices of butterfly abundance implicitly assume that the detectability of individuals within a species remains constant within a site over time and between sites (see [19], [28], [29]). A recent review found that approximately 70% of all published butterfly monitoring studies used counts derived from Pollard walks to estimate population abundance, spatial patterns in abundance, and/or temporal trends in populations [28].

**Figure 1:**
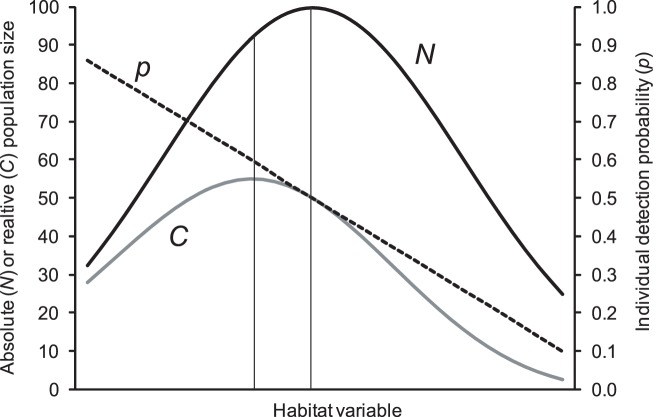
Hypothetical scenario in which a habitat variable influences both absolute population size *N* and also individual detection probability *p*. In this case, changes in the habitat variable (e.g., over time or when comparing different sites) will translate into a divergence of *N* and *C*, the beginning of which is denoted by the vertical bars.

A simple hypothetical scenario will illustrate the problem in failing to account for individual detectability (Figure 1). The abundance of a species changes in relation to some habitat characteristic (illustrated in Figure 1 by a bell-shape curve in abundance *N* over one dimension of the niche, or habitat variable). Some of these habitat characteristics may also influence individual detectability *p* (e.g. sward height, seral stage, canopy cover). If *p* decreases with an increase of the habitat variable, *C* will also decrease, leading to systematically underestimating *N*. In other words, when comparing trends or spatial variation along a habitat gradient, counts will often contradict absolute abundance. In consequence, detectability may be as important in count survey protocols as are the counts themselves. Thus, the meaning of a raw count is hard to evaluate without knowing the associated detection probability.

Estimating true population size requires that the population of interest is subject to a CMR or similar experiment (distance sampling [30]–[32] or replicated counts [33] are alternative techniques). Given the huge effort, capture-mark-recapture experiments are rarely used to compare butterfly true (*N*) and relative (*C*) abundances (e.g. [34], [35]–[40]). Even though all these studies examined the relationships between relative abundance (counts) and absolute population size estimates, none of them looked at *intra*specific variation of individual detectability, which could potentially limit the interpretability of butterfly count data across sites and years. It thus remains largely unclear how counts obtained from Pollard walks and derivatives truly reflect population sizes and trends. This study explores the relationships between absolute and relative population sizes in butterflies and emphasizes that the constant individual detectability assumption can be misleading when studying patterns and trends in butterfly abundance. Therefore we argue that butterfly monitoring programs should systematically assess detectability for at least a subset of sites and years.

## Methods

We studied three butterfly species: the dusky large blue (*Maculinea nausithous*: Lycaenidae), the dryad (*Minois dryas*: Satyridae) and the Iolas blue (*Iolana iolas*: Lycaenidae). All three species are of conservation concern in Switzerland, even though they are not considered as threatened in the European red list of butterflies [41]. For each species, we assessed true population size (*N*) and relative population size (*C*) in contrasting habitats. Each species was surveyed in clearly delimited habitat patches using fixed-route transects representative of the habitat patches of interest, as advocated by Pollard and Yates [14]. All surveyed populations were located in the cantons of Vaud, Fribourg, and Valais in Western Switzerland. Field studies were undertaken with special authorizations from the states of Vaud, Fribourg and Valais wildlife conservation offices.

*M. nausithous* inhabits wet meadows, fens and marshes that support an abundant host plant (*Sanguisorba officinalis*) population. We focused our surveys on two contrasting habitat patches more than 30 km apart: one open fen of approximately 0.5 ha (46°47′23"N 6°40′41"E) and one woodland edge of similar size (46°56′12"N 6°58′45"E). Both patches were clearly delineated by the species’ host plant local distribution. A 700 m zigzagging transect was established across each patch. On 18 days at the fen and 17 days at the woodland edge, an observer and assistant walked each transect at a slow and constant pace. Each observed individual was counted during searches that lasted 60 min on average. Surveys began after 1100 hr and took place on calm weather days following the recommendations of Pollard et al. [12], [14]. Capture-mark-recapture (CMR) was undertaken on the same days as the counts. The sequence in which counts and CMR were undertaken was randomly chosen each day (counts first or CMR first) in order to reduce a potential “trap-shy” response of netted butterflies. CMR took between 60 and 150 min, depending on the number of caught butterflies (Table 1). The surveyed populations of *M. dryas* are localized in wet meadows dominated by *Molinia arundinacea,* the larval host plant, and bushes of *Frangula alnus* and *Berberis vulgaris*. We surveyed two habitat patches as part of a preliminary experiment to test the efficiency of bush removal on *M. dryas*. One of the patches (46°53′56"N 6°55′24"E) was left unmanaged as a control and contained a bush cover of approximately 50%. In the other patch (46°53′53"N 6°55′18"E) the entire bush cover was removed the year prior to surveys. Both habitat patches had a similar size of approximately 2 ha (see [39] for details) and were less than 300 m apart. A 250 m zigzagging transect was established across each patch. Counts were completed (by one observer and one assistant) along each transect on seven days and lasted about 20 min per survey. CMR was undertaken before or after the counts (sequence was chosen at random) and took between 45 and 100 minutes depending on the number of captured individuals. Given the proximity of the two habitat patches, a fraction (7%) of all recaptured individuals had flown from one patch to the other between two subsequent capture occasions.

**Table 1:** Summary of monitoring results.

				Counts		CMR		
		Transect length(m)	Count and CMRsessions	Mean number counted	Maximum count	Total number marked	Recapture fraction	Total estimatedabundance (SE)
*Maculinea nausithous*	Open fen	700	18	17.8	22	97	32%	205 (21)
	Woodland edge	700	17	13.9	23	63	51%	128 (17)
*Minois dryas*	Managed	250	7	35.7	82	238	9%	925 (238)
	Unmanaged	250	7	24.0	57	186	11%	916 (247)
*Iolana iolas*	Bush plantation	40	11	9.7	18	91	40%	92 (1)

*Iolana iolas* is a monophagic lycaenid whose larvae feed exclusively on the bladder senna (*Colutea arborescens*). The Swiss distribution of this butterfly is restricted to planted and natural bushes located on the margins of vineyards in the canton of Valais [42]. We surveyed one of the biggest remnant populations (46°15′51"N 7°24′54"E), which is composed of approximately 20 bushes planted along a vineyard edge [43]. We established a single transect along the entirety (40 m) of this edge. Counts and CMR were undertaken under the same conditions as for the previous two species, with the only difference that a single observer did the counts and the CMR in a random sequence on 11 occasions (days). Counts lasted on average 15 min and CMR lasted between 20 min and 45 min. Summary statistics of all surveyed patches are provided in Table 1.

All captured individuals were numbered with a permanent marker on the underside of the hindwings. This allowed us to create a database of individual capture histories (e.g. 011101) that was used to estimate demographic rates. We used the POPAN parameterization of the Jolly-Seber model [44] implemented in program MARK [45] to estimate demographic parameters (detectability per occasion, recruitment, apparent survival, daily population sizes *N* and total brood size over the season) in all surveyed populations. We tested a set of eight models using either constant or time-specific parameters and compared them using the small-sample corrected Akaike information criterion AICc [46]. The best-performing model for all three species turned out to have constant survival (*φ*), constant detectability per occasion (*p*), and time-specific recruitment (*p_ent_*) and was denoted *φ*(.)*p*(.)*p_ent_*(t) (Table 1). Models using time-varying survival or catchability either did not converge or produced incoherent results (see [38] for a similar example), a byproduct of relatively small capture history matrices (small sample size) and potential overdispersion of the data. For each species and population surveyed, we then estimated *p* by the ratio of *C* on the estimated *N,* for each day.

A generalized estimating equation was then used to compare the individual detectability (slope) between the open fen and woodland edge for *M. nausithous*, and between the managed and unmanaged patch for *M. dryas*. We treated habitat or management status as the factor variable, *N* as the continuous covariate, and *C* as the response. Significant interaction between *N* and the factor variable would suggest a unique detectability for each habitat or management type. The generalized estimating equation was used to account for serial dependence of the repeated observations. We specified the Poisson error distribution and auto-regressive (“AR-1”) correlation structure [47], treating observations within a habitat or treatment as a block of data. We used the geeglm function from the geepack package in RStudio v0.95.262 to specify and fit the model. Correlation between sequential observations in the *M. nausithous* series was 0.242 (0.016 SE) and in the *M. dryas* series 0.138 (0.013 SE), suggesting serial autocorrelation was not strong.

## Results

Individual detectability (*p*) was highly variable, both between and within species (i.e. between sites). Individual detectability in *M. dryas* was as low as 10% whereas in *I. iolas* it was almost ten times higher (97%).

**Figure 2:**
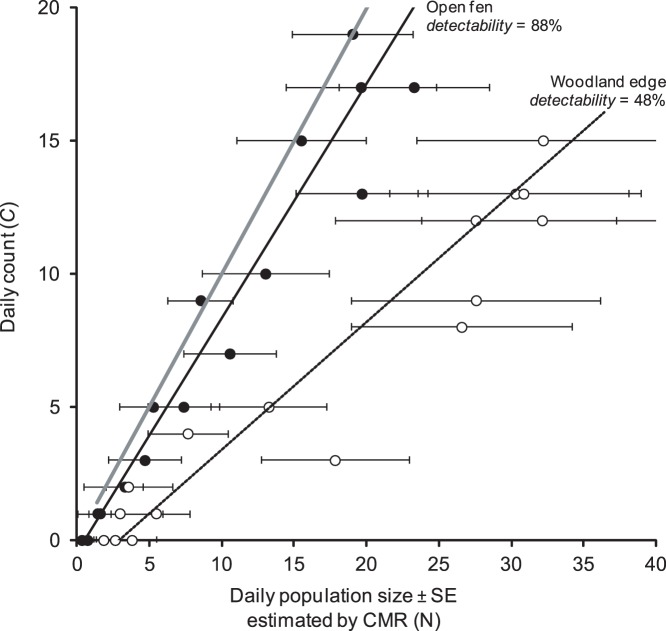
Comparison of daily counts and estimated population size in two populations of the dusky large blue (*Maculinea nausithous*). Closed circles represent fen surveys and open circles represent woodland surveys. The thick grey line indicates the 1∶1 relationship.

Individual detectability in *M. nausithous* varied from 48% to 88% in the woodland edge population and the open fen population, respectively (Figure 2). The difference was statistically significant based on a generalized estimating equation that accounted for serial autocorrelation (Wald statistic  = 3.67e+07, P<0.0001).

**Figure 3:**
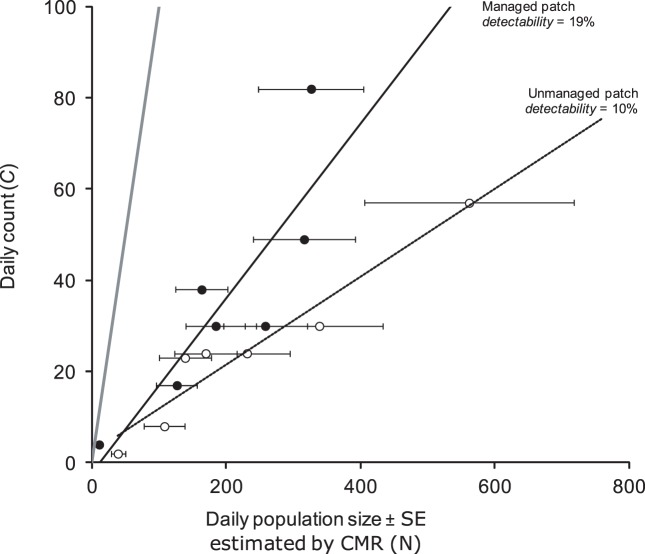
Comparison of daily counts and estimated population size in two populations of the dryad (*Minois dryas*). Closed circles represent the managed patch surveys and open circles represent unmanaged patch surveys. The thick grey line indicates the 1∶1 relationship.

Similarly, individual detectability of the dryad in a managed patch was almost two times higher than in an unmanaged patch (Figure 3). The difference was statistically significant based on a generalized estimating equation that accounted for serial autocorrelation (Wald statistic  = 1.37e+09, P<0.0001).

**Figure 4:**
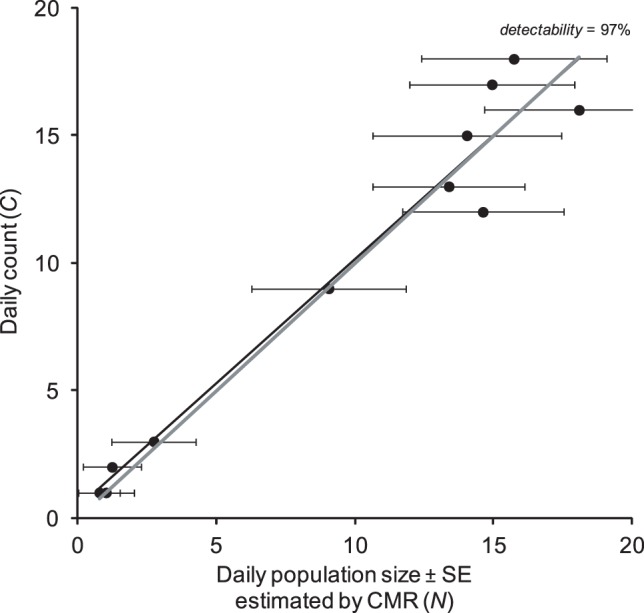
Comparison of daily counts and estimated population size in a population of the Iolas blue (*Iolana iolas*). The slope indicates individual detectability. The thick grey line indicates the 1∶1 relationship.

Our third example suggests that, in some cases, the agreement between counts and estimated population sizes can be extremely high, with an individual detectability close to 1 (97% for *I. iolas*, Figure 4).

## Discussion

This study underscores the need to account for imperfect detection probability in butterfly monitoring programs. The three example species showed a wide range of interspecific detectability (from 0.10 to 0.97). This would probably not come as a great surprise to anyone aware that species can be either cryptic or conspicuous, either due to habitat characteristics (e.g. grassland vs. woodland) or to behavior (e.g. cryptic canopy species vs. riparian patrolling species). Indeed, even the developers of Pollard walks were aware of the fact and cautioned against quantitative comparisons between species [14].

Among our three study species, *M. dryas* is predominantly a percher and hides in dense vegetation during the hottest hours of the day [42], helping explain the low individual detectability. On the other hand, *I. iolas* is fast patrolling and highly territorial around host plant bushes, a conspicuous behavior that accounts for a very high individual detectability. *M. nausithous* has an intermediate behavior, alternating between inconspicuous resting periods and easily observed courtship/mating flights. It perches on the host plant flower heads before engaging into courtship/mating flights with other males flying by.

Habitat patches varied greatly in area (from several square meters for *I. iolas* to approximately 2 ha for *M. dryas*) but we representatively sampled each patch by prorating effort according to patch area. This should have accounted for any area-abundance relationships and differences in the fraction of butterflies observed. It is more likely that butterfly behavior, such as the dichotomy between resting (perchers) and patrolling (fliers), caused differences in detectability between species [20].

Although species-specific behavior can partly explain interspecific variation in detectability, our study more importantly demonstrates that individual detectability also varies *within* a species even under a highly standardized survey protocol. For both *M. dryas* and *M. nausithous*, variation in detectability was attributable to differences in habitat structure. For *M. nausithous*, count data alone would have led us to underestimate the woodland edge population size by about half. As a result, conservation planners may have wrongly assumed the situation is more urgent and taken unnecessary and costly action to acquire more land, plant more nectar species, continue monitoring, etc. Similarly, we have shown that bush removal (as a management action) in *M. dryas* habitat almost doubled individual detectability in the managed patch. This management-induced detectability change is illustrated in Figure 3 (compare the regression slopes). Counts would have led a manager to the conclusion that maximum abundance was higher in the managed patch than in the unmanaged one (peak count and Pollard index higher in the managed patch), when in reality the opposite was true: peak *N* was higher in the unmanaged site. A distance sampling approach [30] applied to these two habitat patches showed the effective strip width to be almost two times higher in the managed patch than in the unmanaged one (see [39] for more details).

Although we could not show that individual detectability increases with increasing population abundance, it could be expected that territorial males might become more mobile, and therefore more detectable, in denser populations. This potential pattern should be more thoroughly researched.

Overlooking intraspecific variance in detection probability in the analysis of monitoring data could be strongly misleading. It is for instance generally accepted that grassland butterflies are widely declining throughout Europe [41], [48] while, at the same time, many agricultural grasslands are abandoned [11], [49]. Although we do not doubt that European grassland butterflies face dramatic and imminent threats, we argue that the observed decline could be due to both a biological process (decrease in habitat quality, leading to a decline in abundance) and to a sampling process (overgrowing of abandoned grasslands leading to a decline of individual detection probability). This line of argument applies to all red-listing efforts. Without incorporating a formal estimation of *p* in monitoring programs, we will not be able to differentiate between the two sources of variation. Is a change in *C* due to changes in *N* or to changes in *p*? Failure to account for variability in *p* could lead to an over-pessimistic red-listing of grassland species [50]. Using a similar logic, we believe that species occupying forest regeneration patches (such as many *Apaturinae* or *Theclinae*) and unmanaged woodland rides and glades will tend to show a strong decline in individual detectability over time as canopy grows and forest mantle becomes less and less visible to the observer (see for instance [37]). Monitoring protocols relying on raw count data are subjected to a presumably strong degree of observational bias in dynamic habitats [19].

Our results lead us to advocate two fundamental changes in butterfly monitoring protocols. The first change is epistemiological: individual detectability should be assumed to be variable, not constant. Because many types of animal studies have found variations in detectability (e.g. [51], [52]), the burden of proof should rest on those who make the constant detectability assumption. The second (and corollary) change is that, resources permitting, estimation of detectability should be explicitly incorporated in survey protocols for most monitoring programs. The only reasonable exception might be for populations of conspicuous species occupying “stable” habitats (e.g. climactic grasslands and forests), or when sampling is exhaustive and in sync with species behavior and life history. One way is to compare count data and absolute population size estimates, as was done here. The most rigorous approach to estimate absolute population size is to conduct a CMR experiment. A CMR directly estimates “catchability”, daily population size, mortality, and brood size [36], [44]. The approach is, however, field-intensive and analytically complex. It also requires large amounts of data, and thus may not work for sparse populations or elusive species where the number of captures (or recaptures) will be too low [34], [38], [53], [54]. CMR may be especially difficult as butterfly handling can lead to increased mortality rates [55], increased emigration rates [34], [54] and changes in activity patterns [56]. As suggested by Murphy [53], it should be used with extreme restraint on small-bodied, swift flying sensitive and/or threatened species. It must however be noted that detectability can be estimated for a subset of sites or years to evaluate whether constant individual detectability may be safely assumed.

Other methods have been developed to assess butterfly population sizes while accounting, directly or indirectly, for detectability, and that do not require handling of animals. Distance sampling is increasingly being used for butterfly abundance monitoring (e.g. [32], [57]) and can be a reliable way to incorporate detectability provided that the main model assumptions are met and the populations are not too sparse [30]. These assumptions can be readily met for some species in some habitats, but recent studies indicate that it cannot be generalized because butterflies tend to gather along linear elements of the landscape (i.e. edges, ecotones… see [8], [32], [58]). Moreover, distance sampling usually requires minimum threshold of 60 observations for accurate modeling and is therefore unlikely to be appropriate for sparse populations [30], [59]. However, hierarchical distance sampling models [60] allow combining estimates from many such sites and should yield improved estimates in sparse data situations.

Royle [33] developed binomial- and N-mixture models for estimating abundance from spatially and temporally replicated counts. Originally designed for bird surveys, this approach is adequate for many monitoring programs in which multiple populations are surveyed repeatedly. This method allows estimating and modeling abundance and detection probability from count data. This class of models enables detectability-corrected abundance estimates in the absence of individual identification. The principal conditions of these models are the temporal replication of counts at a number of sample locations and no double counts. Because this approach assumes that the population is demographically closed between replicated counts (ie. no births, no deaths, no immigrants and no emigrants), repeated butterfly counts must be done within a single day [28], [61]. An interesting feature of this method is that both parameters (abundance *N* and detectability *p*) may be modeled as functions of covariates to increase precision or to investigate covariate relationships. This approach has one major shortfall when applied to butterflies: it requires repeat surveys in a narrow time window, namely multiple surveys on the same day. This may, however, generate little additional cost if counts are repeated by walking back and forth along transects. Such a design may even allow the estimation of butterfly abundance from occupancy data, a shortcut that requires more study [24]. Alternatively, it may be possible in some projects to revisit a subset group of sites several times in the same day and use the data from all sites (including those without replicated counts) in the parameter estimation [62], [63]. Very recent statistical developments allow to relax the closure assumption and to estimate trends in population abundances in a set of populations [64].

Capture-mark-recapture, distance sampling [30], [57] and replicated counts [33] are to our knowledge the only three methods to adequately incorporate detectability in abundance-based survey protocols and trend estimations [64], [65]. Each has advantages and disadvantages and the choice of a method should be based on the behavior and habitat of the study species along with logistic concerns. As recommended by [38], [66] we advocate the application of a limited capture-mark-recapture experiment run in parallel with transect counts whenever a reasonable doubt exists regarding the assumption of constant detectability. This occurs when butterflies inhabit dynamic habitats (either naturally or through management) and in any situation where the probability of detecting an individual is likely to be variable.

Despite obvious flaws, butterfly count-based methods may in some cases provide reliable population estimates (but see [19], [32], [35], [38]). Ernie Pollard provided a brilliant scheme for butterfly monitoring that has been applied for decades in most countries and has served as a basis for estimating species abundance, trends and threat status. However, we believe that recognizing the limitations of raw-count monitoring data is crucial in identifying true patterns and trends in populations. In many cases (as illustrated here), the often invalid assumption of constant individual detectability reduces the interpretability of count data [1], [15], [28], [31]. As evidence-based ecologists and conservationists, our task is to verify our assumptions and, if need be, adapt monitoring protocols to deliver statistically efficient (precise) data and minimally biased inferences on populations. Only such an approach can realistically enable one to act in a cost-effective manner for preserving those segments of biodiversity which are under threat.
